# Editorial: The Effects of the COVID-19 Outbreak on Food Supply, Dietary Patterns, Nutrition, and Health: Volume 1

**DOI:** 10.3389/fnut.2022.845374

**Published:** 2022-02-23

**Authors:** Igor Pravst, Monique M. Raats, Betty Pei Ing Chang

**Affiliations:** ^1^Nutrition and Public Health Research Group, Nutrition Institute, Ljubljana, Slovenia; ^2^Biotechnical Faculty, University of Ljubljana, Ljubljana, Slovenia; ^3^VIST–Faculty of Applied Sciences, Ljubljana, Slovenia; ^4^Food, Consumer Behaviour and Health Research Centre, University of Surrey, Guildford, United Kingdom; ^5^European Food Information Council, Brussels, Belgium

**Keywords:** pandemic, eating behavior, dietary intake, food supply, food safety, food security, food practices, sampling

## Introduction

The COVID-19 coronavirus outbreak has affected populations across the world. In a short time we were exposed to a critical situation and faced with a multitude of medical, social and economic challenges. While the medical community focused on developing successful diagnostic and medical treatments, many countries introduced far-reaching restrictions on daily life to prevent and control the spread of the virus. In many cases, this resulted in a complete lockdown of whole cities, regions, countries, and even countries. The resulting changes to working patterns (e.g., extended working hours, loss of jobs, working from home) and living circumstances have had a large impact on the supply, procurement, preparation, and consumption of food.

Typical measures that governments took to curb the spread of COVID-19 included limiting social and physical interactions, closing schools, hospitality and entertainment venues and other non-critical infrastructure, encouraging people to work from home, limiting the operation of food stores (including the ability for adequate inspection and enforcement activity to take place), and limiting people's ability to leave their home.

Many international borders were closed, which limited the supply of goods, including food. In some areas food supply chains were completely broken or drastically changed with food business operators needing to adopt new business models (e.g., suppliers reorienting themselves to new markets, an increased demand for home delivery). Supplying food also presents possible risks for infection—either environmentally (for example in food stores) or through contaminated foods/packaging. Changes in purchase patterns (e.g., bulk buying of perishable foods that may lead to consumption that is no longer safe or sub-optimal in terms of nutrient content, stockpiling) has led to certain foods being in very limited supply. Increased demand of certain products may result in difficulties in maintaining supply. Communication about food by governments, public health authorities, individual experts and influencers has also increased, using all available media channels and very diverse guidelines.

The measures to prevent and control the spread of COVID-19 and their outcomes have had a profound effect on the food supply, dietary patterns, and nutrition of billions of people. This has given rise to a number of research questions, which are addressed in the Research Topic “Nutrition Eating Behavior Frontiers in Psychology Eating Behavior,” hosted by the Eating Behavior section in Frontiers in Nutrition, and Frontiers in Psychology.

## Pandemic and the Dietary Behaviors

The studies in this issue have all investigated the effect of the pandemic on various aspects of dietary behaviors, using a wide range of methodological approaches. Furthermore, the nature of the pandemic was such that it also affected research methodologies and approaches to data collection. Many countries included lockdowns and self-distancing recommendations, sometimes making on-line studies the only possible option. In some cases, on-line panels were used to assure better representativeness of the study sample, while snow-balling and combinations of different approaches were also used. This editorial presents an overview of these studies, with a particular focus on sampling approaches, and how they were adapted to the circumstances of the pandemic.

### Studies Using Personal Invitations and Face-to-Face Interviews

While the pandemic limited the use of personal approaches to enroll participants, some studies also included face-to-face interviews. Pham et al. investigated determinants of healthy dietary intake and depression on a large sample (*N* = 8,291) of outpatients at 18 hospitals across Vietnam; the data was collected during the waiting time (before/after physical examination). While at the outset interviews were done face-to-face, as the pandemic progressed, self-administered questionnaires were used—either an online version accessible through a QR code, or a printed version. This cross-sectional study was conducted between February and May 2020; collected data included characteristics of patients, health literacy, dietary intake (HES; healthy eating score), and depression (PHQ; patient health questionnaire score; with depression defined as PHQ ≥ 10). Overweight and alcohol consumption were associated with lower healthy eating scores. Furthermore, patients under lockdown with the lowest healthy eating scores had 10-times higher depression odds, while the opposite effect was observed in those with healthier dietary patterns. Higher age, self-employment, lockdown and suspected COVID-19 symptoms were associated with both lower HES scores and likelihood for depression. In contrast, higher education, health literacy, social status and physical activity were identified as protective factors.

### On-Line Studies With a Controlled Sampling Approach

Janssen et al. investigated changes in food consumption during the first COVID-19 lockdown in early 2020. The study was conducted via an on-line survey during the hard lockdown period in three countries—Denmark, Germany, and Slovenia (*N* = 2,680). Quota sampling was used to assure representative age, sex, and regional distribution. The study highlighted that, depending on the type of food, 15–42% of study participants changed their consumption frequency during the pandemic, compared to before. Food categories with the highest change were frozen food, canned food, and cake and biscuits in all three countries. Participants shopped less frequently during lockdown in all three countries, and reported an overall reduction in the consumption of fresh foods, and an increase in the consumption of food with a longer shelf life. The authors further investigated how lockdown measures, pandemic-induced income loss and socio-demographic factors affected changes in consumption patterns. They highlighted a number of differences between the observed countries, such as changes in the consumption of particular food categories from people who had stopped eating in work canteens during the pandemic, which are probably related to the different food cultures.

Similarly, Castellini et al. also used a representative sample of Italian citizens (*N* = 1,004), extracted by stratified sampling through an on-line survey, conducted in May 2020, but their objective was focused on the environmental sustainability of people's diets. Their study revealed that during first COVID-19 phase, about one third of the population reported more frequent consumption of certified sustainable food products, while about 20% indicated an intention to increase such consumption in near future. Researchers highlighted that the psychological impact of the pandemic introduced changes in consumers' attitudes, particularly an increased interest in environmental and health issues.

Bertmann et al. used a Qualtrics on-line panel in a representative state-wide survey in Vermont (US) residents (*N* = 600). They investigated perceptions of food banks and food pantries and their relationship to fruit and vegetable intake and food security in the first half year of the pandemic. Their study results showed more common use of food pantries among households with children and among food insecure households. Respondents from food insecure households not using food pantries repored lower consumption of fruits and vegetables during the pandemic compared to those that did, showing the importance of food banks and pantries for supporting diet quality in at-risk populations during emergency situations.

Clay and Rogus also used sampling with a Qualtrics on-line panel, but with a different sampling approach. Instead of using quotas for population-representative sampling, they used a cross-sectional proportional quota sampling (*N* = 525) to oversample Black, Hispanic, low-income and low-education participants, which were considered to have increased risk for food insecurity and for adverse consequences related to COVID-19. Researchers investigated the relationship between food access concerns, food assistance use, and purchasing behaviors and food insecurity in the state of New York (US) during the pandemic (July–December 2020). The study was conducted using an adapted validated food access survey, developed by the National Food Access and COVID-19 Research Team (NFACT). Higher food insecurity was associated with Hispanic ethnicity, higher food worries, and with the use of food assistance and delivery. The authors concluded that monetary measures would be useful to alleviate barriers to accessing healthy food during pandemic in specific population groups.

Additionally, the pandemic has been shown to be a driver for the increased use of food supplements. For example, in Slovenia Žmitek et al. conducted a repeated cross-sectional study on a representative on-line panel sample (*N* = 835), which showed considerable increase in the use of vitamin D supplements after an educational intervention about the high prevalence of the deficiency during winter time. They further investigated knowledge-related factors of the supplementation. Key predictors for supplementation were knowledge about the health-related impact of vitamin D, dietary sources of vitamin D, and about the widespread prevalence of deficiency in the population. Recommendations for future awareness campaigns were proposed.

While the above-mentioned studies showed the usefulness of the on-line panels for public health research during the pandemic, some researchers used more specific channels in order to target particular populations. For example, Khayyam et al. used specific social platforms (WeChat and QQ) of Pakistani international students to access Pakistani students living in China (*N* = 462) in the wake of the COVID-19 pandemic. The study investigated background factors of food safety and health consciousness in the framework of the theory of planned behavior (TPB) and highlighted the importance of food safety and health consciousness as factors affecting dietary behaviors and intentions.

Somewhat similarly, Bhutani et al. used a specialized channel to recruit participants with a particular profile, but they combined it with a snowball sampling approach. In this cross-sectional study, majority (71%) of the participants (*N* = 1,779) were reached through a Amazon Mechanical Turk platform, enabling the access to younger and underemployed participants with below average incomes. The remainder of the sample were recruited through social media, word of mouth and email invitations. The authors noted that the use of these two methods allowed them to involve a more diverse population. The study was conducted in April/May 2020, and aimed to determine the relationships of health and psychological markers with energy balance-related behaviors during the lockdown. The study highlighted that better food consumption self-control and positive mood were linked with lowering both energy intake and energy expenditure risks. On the other hand, boredom and cravings for sweet and savory foods were linked with higher risk for unhealthy eating and sedentary behavior.

### Studies Using Convenience Sampling, Including Snowball Sampling Approaches

Convenience sampling methods are those that draw on easy to access samples of participants based on location or internet/social media services. These can include snowball sampling where participants are asked to recruit further participants a method that has been used in numerous studies.

De Backer et al. used convenience sampling through social media banners and press releases; their study was conducted in 38 countries, and included 37,207 valid responses. The study was conducted between April and June 2020, and aimed to investigate changes in planning and preparing foods with consideration of personal factors and COVID-19-related social distancing policies. The researchers highlighted that increases in planning/preparing healthy foods were associated with perceived time availability and policies, which caused people to stay at home during pandemic. Financial stress was identified as an important barrier for healthier food choices, highlighting that health inequalities need to be considered very carefully in crisis circumstances.

Within the “International Civil Science Project” Jordan et al. also used international convenience sampling through social media, personal contacts and e-mailing. They investigated changes in the consumption of vegetables during the early pandemic period—April-August 2020, using a semi-structured on-line questionnaire, with about 20% completion rate. Their studied constraints affecting food intake around the world, with particular attention on the consumption of vegetables. The key parameters related to changes in dietary intake were mental stress, home-working, and time spent at home. About a quarter of participants reported changes in the quantity of vegetables and food consumed. The degree of the decrease in the diversity of vegetable intake was commonly linked with sex, occupational and educational status, and household environment. The authors concluded that food systems are subject to prompt pandemic-related transitions, highlighting a need for a strategy that would strengthen the resilience of vulnerable households and support a diverse diet during emergency situations.

Exclusive snowball sampling has been also used in several national studies. Giacalone et al. investigated changes in dietary habits during the COVID-19 lockdown in Denmark, but with a snowball sampling approach. They used an on-line survey, which was distributed to using instant messaging apps e.g., WhatsApp, social media platforms such as Facebook and Twitter, social networking sites such as LinkedIn and ResearchGate and email. The Danish COVIDiet Study had 2,462 respondents (non-representative sample), which reported changes in food intake, focusing on specific food categories that are important in the Mediterranean diet. The study showed a limited effect of the lockdown on dietary habits in the adult population. Very important findings were that many participants reported eating more, snacking more, exercising less, and gaining weight during lockdown, with women generally be more affected than men. The authors highlighted that observed changes are particularly concerning, if sustained for long-term.

Pertuz-Cruz et al. adapted the COVIDiet questionnaire to the dietary behaviors of Colombians, and included 2,745 adults from Colombia using a variety of different channels (social media, conferences, mailings, and press communications) during the first COVID-19 confinement period. The study highlighted several changes in dietary habits, including increased snacking frequency and cooking at home. Overall, a trend toward unhealthier dietary patterns was observed, with notable regional differences.

In Brazil, Liboredo et al. also used snowball convenience sampling to recruit participants during the COVID-19 lockdown. This on-line cross-sectional survey (*N* = 1,368) was conducted between August and September 2020, when Brazil was among the most COVID-19 affected countries in the world. A survey link was distributed through social media, a university website and e-mail invitations. The objective of the study was to investigate the relationship between pandemic related eating behaviors with perceived stress and independently associated factors. Various socioeconomic variables, dietary habits and lifestyle factors were associated with eating behaviors during the quarantine. For example, uncontrolled eating was linked with increased food delivery, while emotional eating was linked with increased food intake and graduation in a non-health-related course.

### Studies Using Sales Data

Sales data were shown to provide very interesting insights into changes in dietary behaviors. For example, Revoredo-Giha and Russo investigated the sale of meats and fish during the first lockdown period in Great Britain. Time sequenced purchases (expenditure and quantities) from a scanner panel dataset, which included about 30,000 households were used to reveal relatively constant proportions of quantities of food. However, the calories provided from saturated fats and the level of sodium in the purchased quantities showed a notable increasing trend, highlighting the reduced nutritional quality of meat/fish purchases in most income groups.

## Pandemic and Food Marketing

The COVID-19 pandemic also affected food supply and marketing, with concerns that food companies could mislead vulnerable populations during a time of increased stress and hardship with increased marketing of unhealthy products. Considering that social media is poorly regulated/controlled and therefore commonly used for the promotion of unhealthy beverages and foods, Gerritsen et al. investigated the social media marketing of foods during the pandemic in New Zealand. Their goal was to assess COVID-washing (specific cause marketing, where companies align themselves with COVID-19 pandemic to enhance their own image) in social media accounts owned by major food and drink brands in the beginning of the pandemic and during lockdown periods (February to May 2020). Public posts from the 20 largest brands were selected for a content analysis, revealing that the majority of brands referenced COVID-19 in posts during the 4-month period, with the peak observed during lockdown. Approximately a quarter (27%) of posts from these brands, particularly fast-food brands, referenced COVID-19. Overall, the study showed that COVID-washing was used to increase brand loyalty and encourage consumption, highlighting that advertising standards should be updated to protect public health.

## Concerns Due to Pandemic-Related Lowering Priorities for the Reduction of Diet-Related Non-Communicable Diseases

In the last 2 years, the COVID-19 pandemic has been taken in center stage—becoming the most important health issue, and pushing aside many other public health matters. As explained by Bösch et al. policymakers have largely ignored non-communicable diseases, a major contributor to mortality, causing 71% of deaths worldwide. It should also be noted that those with NCDs, particularly cardiovascular disease, have been identified as being at high risk for unfavorable COVID-19 outcomes, highlighting that deprioritising NCDs in favor of COVID is not prudent and can have long-lasting negative effects. This deemphasis has resulted in initiatives designed to combat NCDs, such as food reformulation and the elimination of industrially-produced trans fatty acids, being put aside when they should not be.

## Increased Delivery of Food to People's Homes

Several of the above-mentioned studies indicate that food systems are changing quickly and reported an increased use of food delivery services. This trend is logically reflected in increased offers, not only from specialized delivery service providers, but also restaurants adapting their business models to include delivery or pick-up options, when forced to close their dining rooms to be able to continue operating. This challenging situation forced even high-end restaurants to offer home-delivery meals and take-away foods, simply to survive. Spence et al. reviewed approaches to the delivery of high-end dining experience in home environments and the challenges of the explosive pandemic-related growth of this sector. This review highlighted several promising routes for catering providers to optimize multisensory dining experience in people's home.

## Conclusions

The studies in this special issue demonstrate the negative effect of the COVID-19 pandemic on diet and physical and mental health. On average, people's eating behavior was less healthy during the COVID-19 pandemic, particularly during lockdowns, which was in turn associated with weight gain. These studies also shed light on potential mechanisms for this change, such as depression (Pham et al.), reduced frequency of shopping which is associated with a reduction in the consumption of fresh food (Janssen et al.), and the increased marketing of fast food (Gerritsen et al.). Some studies highlighted specific populations that are particularly vulnerable to a decrease in diet quality, including those of a lower socioeconomic status (e.g., Bertmann et al.; Clay and Rogus; De Backer et al.) and those who have NCDs (Bösch et al.). The pandemic further increases the vulnerability of these populations by weakening economic security and food accessibility, and health, respectively. Ironically, the lockdown restrictions that are intended to protect people's health are also those that make it more difficult for them to eat healthily, which in turn can worsen COVID-19 outcomes. This suggests that measures that improve the healthiness of people's diets, such as food banks, financial aid and easier access to fresh food are needed to protect people's health and counter the effects of the pandemic.

## Author Contributions

IP: conceptualization and first draft. BC and MMR: manuscript revision. All authors have read and approved the final version of the manuscript.

## Funding

Preparation of this review was supported by the National Research Program Nutrition and Public Health (P3-0395, funded by the Slovenian Research Agency).

## Conflict of Interest

IP has led and participated in various other research projects in the areas of nutrition, public health, and food technology, which were (co)funded by the Slovenian Research Agency, Ministry of Health of the Republic of Slovenia, the Ministry of Agriculture, Forestry and Food of the Republic of Slovenia, and in cases of specific applied research projects, also by food businesses. BC was employed by European Food Information Council. MMR's research centre has provided consultancy to and received travel funds to present research results from organisations supported by food and drinks companies.

## Publisher's Note

All claims expressed in this article are solely those of the authors and do not necessarily represent those of their affiliated organizations, or those of the publisher, the editors and the reviewers. Any product that may be evaluated in this article, or claim that may be made by its manufacturer, is not guaranteed or endorsed by the publisher.

